# Comparison of intuitive assessment and palliative care screening tool in the early identification of patients needing palliative care

**DOI:** 10.1038/s41598-022-08886-7

**Published:** 2022-03-23

**Authors:** Yung-Feng Yen, Hsiao-Yun Hu, Yun-Ju Lai, Yi-Chang Chou, Chu-Chieh Chen, Chin-Yu Ho

**Affiliations:** 1 Section of Infectious Diseases, Taipei City Hospital, Yangming Branch, Taipei, Taiwan; 2 Institute of Public Health, National Yang-Ming University, Taipei, Taiwan; 3 Department of Health Care Management, National Taipei University of Nursing and Health Sciences, Taipei, Taiwan; 4 Department of Education and Research, Taipei City Hospital, Taipei, Taiwan; 5 University of Taipei, Taipei, Taiwan; 6 School of Medicine, National Yang Ming Chiao Tung University, Taipei, Taiwan; 7 Division of Endocrinology and Metabolism, Department of Internal Medicine, Puli Branch of Taichung Veterans General Hospital, Nantou, Taiwan; 8 Department of Exercise Health Science, National Taiwan University of Sport, Taichung, Taiwan; 9 Department of Family Medicine, Taipei City Hospital, Yangming Branch, Taipei, Taiwan; 10 Department of Psychology, Soochow University, Taipei, Taiwan

**Keywords:** Health care, Risk factors

## Abstract

The intuitive assessment of palliative care (PC) needs and Palliative Care Screening Tool (PCST) are the assessment tools used in the early detection of patients requiring PC. However, the comparison of their prognostic accuracies has not been extensively studied. This cohort study aimed to compare the validity of intuitive assessment and PCST in terms of recognizing patients nearing end-of-life (EOL) and those appropriate for PC. All adult patients admitted to Taipei City Hospital from 2016 through 2019 were included in this prospective study. We used both the intuitive assessment of PC and PCST to predict patients’ 6-month mortality and identified those appropriate for PC. The c-statistic value was calculated to indicate the predictive accuracies of the intuition and PCST. Of 111,483 patients, 4.5% needed PC by the healthcare workers’ intuitive assessment, and 6.7% had a PCST score ≥ 4. After controlling for other covariates, a positive response ‘yes’ to intuitive assessment of PC needs [adjusted odds ratio (AOR) = 9.89; 95% confidence interval (CI) 914–10.71] and a PCST score ≥ 4 (AOR = 6.59; 95%CI 6.17–7.00) were the independent predictors of 6-month mortality. Kappa statistics showed moderate concordance between intuitive assessment and PCST in predicting patients' 6-month mortality (*k* = 0.49). The c-statistic values of the PCST at recognizing patients’ 6-month mortality was significantly higher than intuition (0.723 vs. 0.679; *p* < 0.001). As early identification of patients in need of PC could improve the quality of EOL care, our results suggest that it is imperative to screen patients’ palliative needs by using a highly accurate screening tool of PCST.

## Introduction

Palliative care (PC) services markedly improve the quality of end-of-life (EOL) care in terminally ill patients^1,2^. The World Health Organization (WHO) has estimated that 40 million individuals with cancer and other life-limiting diseases need PC, however only 14% receive it^3^. According to the WHO, PC should be initiated in an early phase of life-limiting diseases and not be restricted to terminal care or the last six to twelve months of life^4^. However, early identification of both patients nearing EOL and those appropriate for PC is a challenge for healthcare systems. Prior reports have shown that clinicians are inaccurate at prognostication and identification of dying patients^5,6^.

Intuitive assessment of PC needs and Palliative Care Screening Tool (PCST) are the tools that are necessary to identify patients nearing the EOL and provide palliative care service for those who need this care^7–9^. An intuitive assessment of PC needs comprises asking the healthcare workers whether a patient will need PC services or whether the patient is going to die within 6–12 months^9^. Since the intuitive assessment of PC needs does not require clinicians to collect patients’ clinical data or use a complex scoring algorithm, it has been widely used in assisting healthcare practitioners in recognizing patients nearing EOL and those appropriate for PC^9^. However, the accuracy of intuitive assessment varies by study population, ranging from a poor to a reasonable accuracy^9^.

The PCST is another assessment method assisting healthcare workers in the early identification of patients nearing EOL and in need of PC^7,8,10^. PCST collects patients’ clinical data (e.g. comorbidities) and uses a scoring algorithm to estimate their period of survival^8,10^. Although intuitive assessment of PC and PCST are the assessment tools assisting healthcare providers in identifying patients appropriate for PC, the comparison of their prognostic accuracies remains unexplored. A previous Australian cohort study followed 4365 patients aged 70 years and over to compare the validity of intuition and the Supportive and Palliative Care Indicators Tool (SPICT) in predicting their mortality^11^. This study showed that the SPICT had a higher sensitivity but a lower specificity in predicting patients’ 12-month mortality, compared to intuition^11^.

Determining the validity of intuitive assessment and PCST for early identification of patients nearing EOL could inform future PC policies and practices to guide accurate screening for patients’ needs and improve the scope of PC services. The goal of this cohort study was to compare the prognostic accuracy of intuitive assessment and PCST in the early identification of patients needing PC.

## Methods

### Background information and study participants

Taipei City Hospital (TCH), a 4700-bed facility, is the largest healthcare organisation in Northern Taiwan. TCH, since 2015, has initiated a large-scale palliative care program to facilitate early identification of patients in need of PC and to provide EOL discussions for the patients^12^. When patients are admitted to TCH, their PC needs were evaluated by the primary care nurses using the PCST (as shown in Supplementary Table 1)^13^ and intuitive assessment of PC needs. The PCST is a one-page screening tool adapted from the instrument used in St. Mary’s medical center and Center to Advance Palliative Care in the United State^13^. The total score of PCST ranges from 0 to 31. If patients’ PCST score is 4 points or greater, they are informed about EOL discussions to discuss their preference regarding EOL care. To promote EOL discussions to patients nearing death, EOL discussions are also initiated for patients whose PC needs are ascertained through intuitive assessment by healthcare workers. During the EOL discussions, healthcare providers discuss patients’ goals and preferences concerning medical care towards the end of their lives.

This study included participants who were aged 18 years or older and were admitted to TCH from 2016 through 2019. All patients were followed up until December 31, 2020 or their death. The Institutional Review Board of TCH (no. TCHIRB-11003001-E) approved this study. The informed consents for study participants were waived in this report. All methods in this study were performed in accordance with relevant guidelines and regulations. This study is also in accordance with the Declaration of Helsinki.

### Palliative care training program for healthcare providers

TCH, since 2015, has held a series of 26 palliative training programs for all healthcare providers in order to early identify patients nearing the EOL and provide PC services to those who need them^14^. A total of 2820 healthcare providers have completed the palliative training programs, including 619 (22.0%) physicians, 1128 (40.0%) nurses, and 76 (2.7%) social workers. These palliative training programs include: (1) early identification of patients near death and those in need of PC, (2) education regarding PC for patients with terminal illnesses (3) training in communication skills with patients during the EOL discussions, and (4) training in providing spiritual support for patients and their families. The training program includes 39 h of didactic training and 4 h of simulation training on EOL discussions. All healthcare providers were encouraged to receive the palliative training program before conducting the EOL discussions with patients.

### Outcome variable

The primary outcome of this study was the 6-month mortality risk of patients. Deaths were determined by patients’ medical records.

### Main explanatory variable

The main explanatory variables were the intuitive assessment of PC needs and PCST score. When patients are admitted to TCH, their primary care nurses used intuitive assessment to evaluate their palliative needs through the question, ‘Would I agree that this patient needs palliative care services?’ The responses to the intuitive assessment of PC needs included either ‘yes’ or ‘no’. PCST scores were categorised into < 4 or ≥ 4 points. Our prior validity study found that the specificity of a PCST score ≥ 4 in predicting 6-months mortality was 91.8% among hospitalised patients^13^.

### Controlling variables

The controlling variables included sociodemographics, hospital units, and comorbidities. The sociodemographic characteristics included age and gender. Hospital units were categorized as general ward and intensive care unit. Comorbidity was determined using the patients’ medical records. The comorbidities included cancer, heart failure, chronic obstructive pulmonary disease (COPD), liver cirrhosis, end-stage renal disease, and cerebrovascular accident.

### Statistical analysis

First, we analyzed the subjects’ demographic data. We then presented continuous data as mean ± standard deviation (SD), and conducted a two-sample *t* test to compare outcomes between patients who needed PC services and those who did not. We analyzed categorical data using the Pearson χ^2^ test where appropriate.

We used Kaplan–Meier method to generate survival curves, with comparisons being evaluated according to the intuitive assessment of PC needs and PCST scores of patients. Multivariate logistic regression was used to estimate the association of intuitive assessment and PCST scores with 6-month mortality after adjusting for participants’ age, gender, and comorbidities. The variable with *p* < 0.05 was defined as a significant factor associated with the outcomes in the multivariate analysis. Adjusted odds ratios (AOR) with 95% confidence intervals (CI) were reported to show the strength and direction of these associations.

We used 2 × 2 contingency tables to calculate the concordance between intuitive assessment and PCST scores in predicting patients' 6-month mortality^15^. The strength of this agreement was assessed using the kappa (*k*) statistic.

To assess the prognostic prediction of intuitive assessment and PCST scores, we calculated the specificity (the ability to recognize those not nearing the EOL), sensitivity (the ability to recognize patients nearing the EOL), positive predictive value (PPV; the proportion of patients who died when the healthcare providers predicted nearing the EOL), and the negative predictive value (NPV; the proportion of patients who survived when the healthcare providers predicted survival)^16^. We estimate the c-statistic value, also known as the area under the curve, to indicate the level of predictive accuracy of intuition and PCST^17^. A score of 0.5 suggests a model with poor predictive value, meaning that healthcare providers are no better than chance at identifying a patient nearing EOL. An increase in the c-statistic value indicates an increase in the level of predictive accuracy. A good predictive model requires the c-statistic score to be > 0.7. The difference of c-statistic value for intuitive assessment and PCST was compared from different logistic regression models^18^. All data management and analyses in this study were performed using the SAS 9.4 (SAS Institute, Cary, NC) and STATA 13.0 (STATA Corp, College Station, TX) software package.

## Results

### Participant selection

This cohort study included 130,361 patients who were admitted to TCH and were evaluated for PC needs, from 2016 through 2019. After excluding those younger than 18 years (*n* = 18,878), the remaining 111,483 patients were included in the analysis. The overall mean (SD) age was 60.9 (19.1) years; and 50.6% of the subjects were male. Of all study subjects, 4,984 (4.5%) individuals needed PC services based on intuitive assessment by healthcare workers, and 7428 (6.66%) subjects obtained a PCST score ≥ 4.

### Characteristics of patients evaluated by intuitive assessment of palliative care needs

Table 1 shows the characteristics of patients according to intuitive assessment of PC needs. Compared with patients without PC needs, those with PC needs were older (76.3 vs 60.2 years). Moreover, patients with PC needs showed higher proportions of comorbidities and were more likely to be admitted to the intensive care unit. The proportion of patients with PCST scores ≥ 4 were 64.29% and 3.97% in those with and without PC needs, respectively. Additionally, 45.12% of patients with PC needs had undergone EOL discussions with physicians, with only 5.07% of patients without PC needs receiving the same discussions.

**Table 1:** Patients’ characteristics according to intuitive assessment of palliative care needs. SD, standard deviation; COPD, chronic obstructive pulmonary disease; EOL, end-of-life. *Unless stated otherwise.

Characteristics	No. (%) of subjects*			*p* value
	Total, n = 111,483	Palliative care needs ‘yes’, n = 4984	Palliative care needs ‘no’, n = 106,499	
**Age, yrs**				
Mean ± SD	60.9 ± 19.1	76.3 ± 15.4	60.2 ± 19.0	< .001
18–64	61,393 (55.07)	1141 (22.89)	60,252 (56.58)	< .001
≥ 65	50,090 (44.93)	3843 (77.11)	46,247 (43.42)	
**Sex**				
Female	55,126 (49.45)	2379 (47.73)	52,747 (49.53)	0.013
Male	56,357 (50.55)	2605 (52.27)	53,752 (50.47)	
**Hospital units**				
General ward	110,070 (98.73)	4536 (90.01)	105,534 (99.09)	< .001
Intensive care unit	1413 (1.27)	448 (8.99)	965 (0.91)	
**Comorbidities**				
Cancer	9479 (8.50)	1162 (23.31)	8317 (7.81)	< .001
Heart failure	4260 (3.82)	538 (10.79)	3722 (3.49)	< .001
COPD	3340 (3.00)	362 (7.26)	2978 (2.80)	< .001
Liver cirrhosis	1220 (1.09)	124 (2.49)	1096 (1.03)	< .001
End-stage renal disease	89 (0.08)	12 (0.24)	77 (0.07)	< .001
Cerebrovascular accident	8083 (7.25)	552 (11.08)	7531 (7.07)	< .001
**Palliative Care Screening Score**				
< 4 points	104,055 (93.34)	1780 (35.71)	102,275 (96.03)	< .001
≥ 4 points	7428 (6.66)	3204 (64.29)	4224 (3.97)	
EOL discussions	7651 (6.86)	2249 (45.12)	5402 (5.07)	< .001
**Outcome**				
Death within 180 days of palliative care screening	3978 (3.57)	1549 (31.08)	2429 (2.28)	< .001
Death during the study follow-up period	7297 (6.55)	1904 (38.20)	5393 (5.06)	< .001

### Mortality rates by intuitive assessment of palliative care needs and PCST

**Figure 1:**
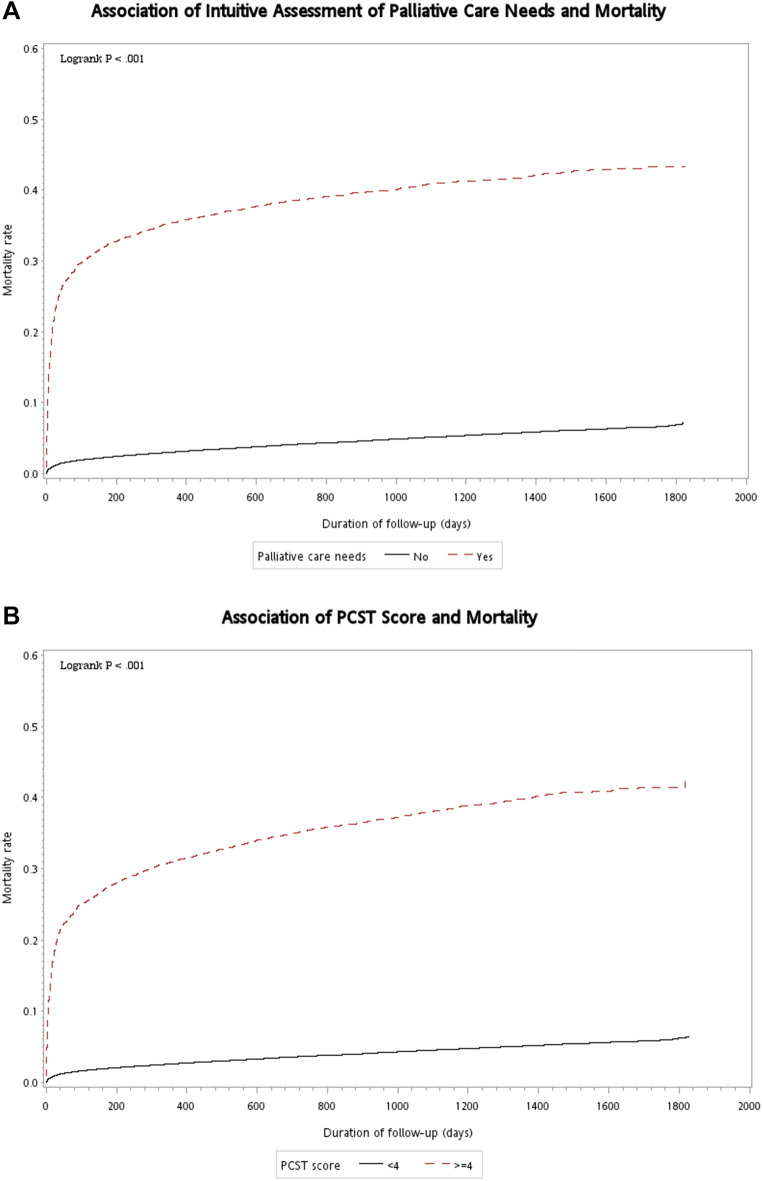
(**A**) Kaplan–Meier curves for time to death in patients with an intuitive assessment of palliative care needs ‘yes’ and ‘no’. (**B**) Kaplan–Meier curves for time to death in patients with PCST scores of ≥ 4 and < 4. Abbreviations: PCST: palliative care screening tool.

During the follow-up period, 3,978 patients died within 6 months of palliative care screening, including 1549 (31.08%) individuals with PC needs and 2429 (2.28%) patients without PC needs. The proportion of 6-month mortalities were 26.56% and 1.92% in patients with PCST scores of ≥ 4 and < 4, respectively. Time to death was significantly shorter in patients with PC needs when compared to those without PC needs (*P* < 0.001, log-rank test; Fig. 1A). When compared with patients with PCST scores of < 4, the mortality rate was significantly higher among those with scores ≥ 4 (*P* < 0.001, log-rank test; Fig. 1B).

### Factors associated with 6-month mortality among patients who received palliative care screening

Multivariate logistic regression was used to identify the independent risk factors for 6-month mortality in patients assessed for PC needs. After controlling for demographics and comorbidities, patients with PC needs showed a significantly higher risk of 6-month mortality compared to those without PC needs (AOR = 9.89; 95% CI 9.14–10.71; *P* < 0.001) (Table 2). Moreover, a PCST score ≥ 4 was associated with a higher risk of 6-month mortality (AOR = 6.59; 95% CI 6.17–7.00; *p* < 0.001). The independent risk factors for 6-month mortality were age ≥ 65 years, being male, admission to intensive care unit, cancer, heart failure, COPD, liver cirrhosis, and cerebrovascular accident.

**Table 2:** Univariate and multivariate analyses of factors associated with 6-month mortality among patients. ^***^ < .001. AOR, adjusted odds ratio; CI, confident interval; COPD, chronic obstructive pulmonary disease.

Variables	Number of patients	6-month mortality	Univariate	Multivariate analysis	
		n (%)	OR (95% CI)	Model 1, AOR (95% CI)	Model 2, AOR (95% CI)
**Palliative care needs**					
No	106,499	2429 (2.28)	1	1	
Yes	4984	1549 (31.08)	19.32 (17.97–20.77)***	9.89 (9.14–10.71)***	
**Palliative Care Screening Score**					
< 4 points	104,055	2005 (1.98)	1		1
≥ 4 points	7428	1973 (26.56)	18.41 (17.20–19.70)***		6.59 (6.19–7.00)***
**Age, yrs**					
18–64	61,393	743 (1.21)	1	1	1
≥ 65	50,090	3235 (6.46)	5.64 (5.20–6.11)***	3.83 (3.51–4.18)***	4.90 (4.58–5.24)***
**Sex**					
Female	55,126	1701 (3.09)	1	1	1
Male	56,357	2277 (4.04)	1.32 (1.24–1.41)***	1.31 (1.22–1.41)***	1.27 (1.20–1.34)***
**Hospital units**					
General ward	110,070	3486 (3.17)	1	1	1
Intensive care unit	1413	492 (34.82)	16.33 (14.57–18.32)***	9.79 (8.53–11.24)***	3.82 (3.35–4.36)***
**Comorbidities**					
Cancer	9479	1315 (13.87)	6.01 (5.60–6.44)***	5.32 (4.91–5.77)***	4.56 (4.27–4.86)***
Heart failure	4260	485 (11.38)	3.82 (3.45–4.22)***	2.53 (2.25–2.84)***	2.34 (2.13–2.56)***
COPD	3340	251 (7.51)	2.28 (1.99–2.60)***	1.33 (1.14–1.54)***	1.74 (1.57–1.94)***
Liver cirrhosis	1220	163 (13.36)	4.30 (3.64–5.09)***	2.25 (1.85–2.73)***	2.57 (2.20–3.01)***
End-stage renal disease	89	19 (21.35)	7.37 (4.43–12.24)***	4.89 (2.67–8.98)***	3.05 (1.75–5.32)***
Cerebrovascular accident	8083	380 (4.70)	1.37 (1.23–1.52)***	1.24 (1.10–1.40)***	1.15 (1.05–1.26)***

### Correlation between intuitive assessment and PCST in predicting patients' 6-month mortality

Kappa statistics showed moderate concordance (*k* = 0.49) between intuitive assessment and PCST in predicting patients' 6-month mortality (Table 3). Patients with both PCST score ≥ 4 and PC needs reported as ‘no’ were major contributors to the discrepancy between intuitive assessment of PC needs and PCST in predicting patients' 6-month mortality.

**Table 3:** Agreement between intuitive assessment of palliative care needs and PCST in predicting patients' 6-month mortality. PCST, Palliative Care Screening Score.

	Palliative care needs ‘yes’	Palliative care needs ‘no’	Total n	Agreement %	*k*	*P value*
PCST score < 4	1780	102,275	104,055			
PCST score ≥ 4	3204	4224	7428			
n	4984	106,499	287	94.6	0.49	< .001

### Accuracy of intuitive assessment of palliative care needs and PCST in predicting patients’ 6-month mortality

Table 4 shows the accuracy of the intuitive assessment of PC needs and PCST at predicting 6-month mortality in patients. The c-statistic values of the intuitive assessment of PC needs and PCST at recognizing patients in last 6 months of life were 0.679 and 0.723, respectively (*p* < 0.001).

**Table 4:** Accuracy of intuitive assessment of palliative care needs and PCST in predicting patients' 6-month mortality. CI, confident interval.

	Sensitivity % (95% CI)	Specificity	Positive predictive value	Negative predictive value	C-statistic
Intuitive assessment of palliative care needs	38.9 (37.4–40.5)	96.8 (96.7–96.9)	31.1 (30.0–32.2)	97.7 (97.6–97.8)	0.679
Palliative care screening tool	49.6 (48.0–51.1)	94.9 (94.8–95.1)	26.6 (25.8–27.4)	98.1 (98.0–98.2)	0.723

## Discussion

In this cohort study of 111,483 patients, 3978 (3.57%) individuals died within 6 months of palliative care screening. After adjusting for demographics and comorbidities, a positive response ‘yes’ to intuitive assessment of PC needs and a PCST score ≥ 4 were found to be the independent predictors for patients’ 6-month mortality. Additionally, Kappa statistics showed moderate concordance between intuitive assessment and PCST in predicting patients' 6-month mortality. A comparison of prognostic accuracy of intuitive assessment and PCST indicated that PCST was better at predicting 6-month mortality in patients than intuitive assessment.

Early identification of patients nearing EOL is an important step to deliver PC services to those who need such care^19^. Intuitive assessment and PCST are the screening tools assisting healthcare providers in predicting patient outcomes and early identification of those in need of PC^7,9^. However, the comparison of the validity of intuitive assessment and PCST has not been extensively studied. An Australian study involving 4365 patients found that PCST was better at predicting 12-month mortality than intuitive assessment (1.6% vs. 1.1%) although with no significant difference between these two tools^11^. Our study followed 111,483 patients and found that the prognostic accuracy of PCST was significantly better than intuitive assessment (72.3% vs. 67.9%). As early identification of people nearing EOL could create greater opportunities for providing PC services, as well as improve their quality of EOL care^1^, our study suggests that it is important to screen patients’ palliative needs via an accurate screening tool.

This study found that 33.6% of deceased patients underwent EOL discussions with physicians before their death, which was higher than 31.2% among deceased cancer patients in the US^20^. The palliative program to promote EOL discussions at Taipei City Hospital may account for the high proportion of Taiwanese patients undergoing EOL discussions nearing the end of their lives. The Taipei City Hospital, since 2015, has initiated a large-scale PC program to early identify patients nearing the EOL^12^. A palliative care screening tool was embedded into the existing electronic medical healthcare system to evaluate patients’ PC needs. The duration of completing an electronic PCST assessment for each patient was about five minutes. Patients with a PCST score ≥ 4 or a positive response ‘yes’ to an intuitive assessment of PC needs were informed about EOL discussions to determine their preference regarding EOL care. During the EOL discussions with physicians, patients’ expectations and goals regarding the EOL care were discussed and emphasized. Since EOL discussions improved patient quality of care^21,22^, the findings of our study suggest that it is important to incorporate a comprehensive palliative care screening tool into the existing healthcare system to early identify hospitalized patients nearing EOL and provide EOL discussions to better align care with their preferences.

Palliative programs for early identification of patients nearing EOL are less common in the regions of Asia^23^. Taiwan has been a leader in Asia with regard to palliative care^24^ and launched the first Patient Self-Determination Act (PSDA) in 2015^25^. Since a sustainable palliative program service should include all health care professionals across different specialty who are able to provide a palliative care approach for patients with serious illnesses^26,27^, Taipei City Hospital, since 2015, has held a series of palliative training programs and trained all healthcare providers in the provision of PC and EOL discussions for patients^14^. Although integrated PC programs have been seldom implemented in Asia^28^, our study demonstrated that it is feasible to implement a comprehensive palliative program for early identification of patients needing PC and promote EOL discussions for the patients.

There are two strengths in our study. First, our cohort study was a large-scale study comparing the validity of intuitive assessment and PCST to recognize patients nearing the EOL. Our study found that the accuracy of PCST was better than intuitive assessment in predicting patients’ 6-month mortality. Since early identification of patients nearing EOL could facilitate more effective palliative care planning, our study suggests that early identification using an accurate screening tool is imperative. Second, although palliative programs for early identification of patients in need of PC are uncommon in Asia^23^, our study suggests that it is practical to incorporate a palliative screening program into the existing healthcare system to ensure early identification of patients in need of PC.

Nonetheless, one limitation should be considered in interpreting our findings. The external validity of our findings may be a concern because almost all our enrolees were Taiwanese. The generalizability of our results to other, non-Asian ethnic groups thus requires further verification.

In summary, this population-based cohort study found that an intuitive assessment of PC needs with a positive response ‘yes’ and a PCST score ≥ 4 were the independent predictors for 6-month mortality in patients. There was a moderate concordance between intuitive assessment and PCST in predicting patients' 6-month mortality. In terms of prognostic accuracy, PCST was better than intuitive assessment in predicting patients’ 6-month mortality. As the early identification of patients in need of PC could help in effectively meeting their treatment goals and improve the quality of EOL care, it is imperative to screen patients’ palliative needs by using a highly accurate screening tool.

## Supplementary Information

Supplementary Information.
