# Supplementary material for: Comparison of intuitive assessment and palliative care screening tool in the early identification of patients needing palliative care
Source: Sci Rep. 2022 Mar 23;12:4955. doi: 10.1038/s41598-022-08886-7 (PMC8943025; doi:10.1038/s41598-022-08886-7)
Supplement: Supplementary file 1 — Supplementary Information. [file 41598_2022_8886_MOESM1_ESM.docx]

| Screening Items | Scoring |
| --- | --- |
| A. Primary diseases associated with palliative care | Score 2 points EACH |
| 1. Cancer (metastatic/recurrent) |  |
| 2. Severe chronic obstructive pulmonary disease (COPD) |  |
| 3. End-stage liver disease (e.g., hepatoencephalopathy, severe ascites, gastroesophageal variceal hemorrhage) |  |
| 4. End-stage renal disease (age ≥65, dialysis for >2 years) |  |
| 5. Severe heart failure (shortness of breath at rest or orthopnea) |  |
| 6. Neurologic disease with severely reduced function (i.e. dementia, stroke, coma resulting in bedridden status) |  |
| 7. Other life-limiting acute illness (i.e. ARDS, sepsis, multiple organ failure) |  |
|  |  |
| B. Secondary comorbidities | Score 1 point EACH |
| 1. Cancer (primary) |  |
| 2. Moderate COPD |  |
| 3. Liver cirrhosis |  |
| 4. End-stage renal disease (others) |  |
| 5. Moderate heart failure (Dyspnea on exertion) |  |
| 6. Other complex diseases (e.g., chronic wound, catastrophic illness, multiple trauma) |  |
|  |  |
| C. Functional status of patient | Score as specified |
| Using ECOG Performance Status (Eastern Cooperative Oncology Group) |  |
| Score Scale |  |
| 1. Fully active, able to carry on all pre-disease activities   without restrictions. |  |
| 1. Restricted in physically strenuous activity but   ambulatory and able to carry out work of a light or sedentary nature, e.g., light housework, office work. |  |
| 1. Ambulatory and capable of all self-care but unable to   carry out any work activities. |  |
| 1. Capable of only limited self-care; confined to bed or   chair |  |
| 3 Completely disabled. Cannot carry on any self-care. |  |
|  |  |
| D. Other criteria to consider when screening the patient, including frequency of disease exacerbations: | Score 1 point EACH |
| 1. The patient/family needs help when making complex decisions and determining the goals of patient care |  |
| 2. The patient suffered from intolerable pain |  |
| 3. The patient had unsolved psychosocial or spiritual issues |  |
| 4. The patient visited the Emergency Department more than once for the same diagnosis in the last 30 days |  |
| 5. The patient was hospitalized more than once for the same diagnosis in the last 30 days |  |
| 6. The patient had ever admitted into the intensive care unit during hospitalization |  |
| 7. The patient was admitted into the intensive care unit with poor prognosis |  |
| 8. The patients was hospitalized for more than 30 days. |  |
| Total Score: A + B + C + D= |  |

**Supplementary table 1.** Palliative care screening tool (PCST).
